# Review of Myocardial Ischemia, Scar, and Viability Estimation with Molecular Magnetic Resonance Imaging

**DOI:** 10.3390/biomedicines12081681

**Published:** 2024-07-27

**Authors:** Saara Sillanmäki, Suvi Hartikainen, Elias Ylä-Herttuala

**Affiliations:** 1 Institute of Clinical Medicine, University of Eastern Finland, 70029 Kuopio, Finland; 2 Diagnostic Imaging Center, Kuopio University Hospital, 70200 Kuopio, Finland; 3 A.I. Virtanen Institute for Molecular Sciences, University of Eastern Finland, 70211 Kuopio, Finland

**Keywords:** molecular imaging, ischemia, magnetic resonance imaging, myocardial infarction, ischemic heart disease, viability

## Abstract

Background: Cardiovascular diseases, particularly myocardial ischemia from coronary artery obstruction, remain a leading cause of global morbidity. This review explores cardiac molecular magnetic resonance imaging (mMRI) and other molecular imaging techniques for the evaluation of myocardial ischemia, scarring, and viability. Results and findings: mMRI imaging methods provide detailed information on myocardial ischemia, edema, and scar tissue using techniques like cine imaging, T1 and T2 mapping, and gadolinium-based contrast agents. These methods enable the precise assessment of the myocardial tissue properties, crucial in diagnosing and treating cardiovascular diseases. Advanced techniques, such as the T1ρ and RAFFn methods, might provide enhanced contrast and sensitivity for the detection of myocardial scarring without contrast agents. Molecular probes, including gadolinium-based and protein-targeted contrast agents, improve the detection of molecular changes, facilitating early diagnosis and personalized treatment. Integrating MRI with positron emission tomography (PET) combines the high spatial and temporal resolution with molecular and functional imaging. Conclusion: Recent advancements in mMRI and molecular imaging have changed the evaluation of myocardial ischemia, scarring, and viability. Despite significant progress, extensive research is needed to validate these techniques clinically and further develop imaging methods for better diagnostic and prognostic outcomes.

## 1. Introduction

Cardiovascular diseases persist as a predominant cause of morbidity on a global scale [1]. Myocardial ischemia, which typically results from coronary artery obstruction, can lead to various conditions, such as myocardial infarction and reversible ischemic states like hibernation or stunning. Inflammation plays a crucial role in cardiac healing via the removal of dead cells and the formation of scar tissue [2]. Over time, it may contribute to cardiac remodeling, possibly further leading to adverse cardiovascular consequences like heart failure (HF). This cascade can be visualized using molecular imaging techniques, including cardiac molecular magnetic resonance imaging (mMRI). One of the key advantages of magnetic resonance imaging (MRI) is its high spatial resolution and soft tissue contrast, allowing for the simultaneous evaluation of the cardiovascular anatomy, physiology, and molecular events without a radiation burden [3]. This review aims to summarize the current knowledge on the application of mMRI and other molecular imaging techniques in the evaluation of myocardial ischemia, scarring, and viability. By synthesizing recent advancements and highlighting their clinical implications, this review seeks to provide a comprehensive resource for clinicians and researchers.

## 2. Current Guidelines for Diagnostics and Treatment

The basic diagnostic methods for coronary artery disease (CAD) include electrocardiography, echocardiography, noninvasive imaging such as computed tomography angiography, nuclear medicine methods (single-photon emission computed tomography and positron emission tomography (PET)), invasive coronary angiography, and sometimes cardiac MRI. The current treatment recommendations for ischemic cardiovascular disease aim to reduce the risk of heart attacks and manage symptoms [4].

Key treatment strategies for CAD include controlling high blood pressure, high cholesterol, and diabetes; quitting smoking; maintaining a healthy diet; and engaging in regular physical activity [5]. Medications such as aspirin, statins, and beta-blockers, as well as sodium-glucose cotransporter 2 inhibitors and glucagon-like peptide-1 receptor agonists, may be necessary [5]. In severe cases, revascularization may be considered [5].

## 3. Biological Background of MRI Imaging for Cardiac Ischemia, Scarring, and Viability

**Figure 1:**
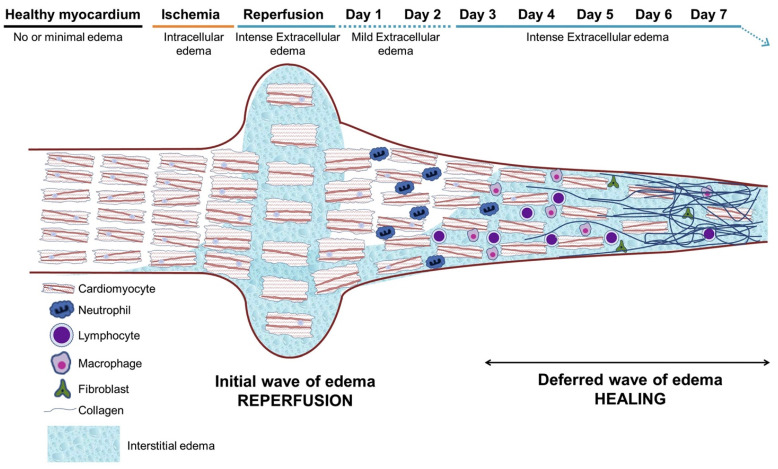
The dynamic tissue composition changes after ischemia and reperfusion. Image adapted from Ibanez et al., 2019 [8] by CC BY-NC-ND 4.0. https://creativecommons.org/licenses/by-nc-nd/4.0/.

Cardiovascular risk factors promote changes in endothelial shear stress, which leads to endothelial dysfunction and chemokine secretion [6]. This leads to the overexpression of leucocyte adhesion molecules, which recruit monocytes to the area [7]. The inflammatory response to atherosclerosis serves as an imaging target, as either antibodies against adhesion molecules or microparticles that are phagocyted into macrophages. An unstable or fragile plaque is characterized by a thin fibrous cap with signs of inflammation and a dense lipid core. Damage to the fibrous cap leads to thrombus formation. As the thrombus grows, it quickly occludes the artery and causes myocardial ischemia. During ischemia, slight intracellular edema can be detected [8]. After an infarction, part of the myocardium may be ischemic yet still viable. In this case, the function may be preserved or restored by spontaneous or medically induced revascularization [9]. During reperfusion, rapid and intense extracellular edema formation is prevalent, resulting in myocardial swelling (Figure 1). Inflammatory cells infiltrate the ischemic region and the progressive replacement of cardiomyocyte debris with collagen and an extracellular matrix occurs (Figure 1). In the secondary phase, swelling occurs in the myocardium (Figure 1), lasting days or weeks depending on the remodeling processes. In the healing process, the cardiomyocytes are replaced by an extracellular matrix (e.g., collagen), leading to the shrinkage of the myocardial thickness [8].

## 4. Principles of Molecular Imaging in Cardiac MRI

MRI is a versatile tool used to evaluate the cardiac anatomy, function, and myocardial tissue properties across various cardiovascular diseases. Moreover, MRI can offer detailed information on myocardial ischemia, edema, scarring, and tissue viability (Table 1). It is currently regarded as the gold standard in determining the myocardial anatomy and function, offering a high spatial resolution [10,11,12].

### 4.1. Cardiac Anatomy and Function

The cardiac anatomy and function can be assessed using contrast agent (CA)-free cine imaging. These images enable an accurate assessment of the end-diastolic volume (EDV) and end-systolic volume (ESV) by delineating the borders of the epicardium. Using these volumes, metrics such as the ejection fraction (EF), stroke volume (SV), and cardiac output (CO) can be calculated [10,11,12]. These parameters are used to diagnose various cardiovascular diseases, including heart failure (HF) [10,11,13,14]. Additionally, these cine images allow us to measure the myocardial thickness, which varies during the ischemic and scarring process [13,14].

Cine images are typically obtained using a gradient echo-based imaging sequence, which captures images of the heart over more than 95% of one heart cycle [15]. Both bright-blood (BB) and dark-blood (DB) imaging techniques can be used to determine the cardiac anatomy, function, ischemia, and scarring [16,17]. Texture analysis, such as T1 and T2 mapping, has been developed to evaluate the cardiac tissue properties by identifying patterns and relationships between neighboring pixels [18]. This technique helps to differentiate between viable, ischemic, and scarred areas in the myocardium [18].

There are also several advanced techniques for myocardial motion analysis, including tagging, displacement encoding with stimulated echoes (DENSE), and feature tracking, which further enhance the assessment of cardiac function [19,20]. While strain values are typically derived from echocardiography, they can also be obtained through MRI using techniques such as feature tracking and DENSE. The strain is typically estimated in the longitudinal, radial, or circumferential directions. For example, the longitudinal strain reflects the deformation of the myocardium along the length of the heart from the base to the apex. Strain imaging is effective in detecting subtle changes in myocardial function. It is known to help to detect ischemia, scarring, and the viable myocardium [21,22,23].

### 4.2. Contrast Agent for Myocardial Ischemia, Scar, and Viability Imaging

Ischemia and scar tissue can be effectively identified using MRI with the aid of a CA administered during the imaging process. Gadolinium (Gd) is the most used CA in MRI for the diagnosis and evaluation of various cardiovascular diseases in clinical practice. Gadolinium-based contrast agents (Gd-CA) accumulate in the expanded extracellular space of damaged tissue, such as ischemic and scar tissue. In contrast, in the normal myocardium, the Gd atoms are quickly washed out, which can be seen as lower contrast. This differential accumulation allows for the visualization of damage from healthy myocardial tissue [10,24]. The Gd is a paramagnetic atom, which means that it reacts on the external magnetic field and, therefore, it creates a hyperintensity area in T1-weighted MR images where Gd atoms have gathered [24].

Most clinical cardiac MRI protocols include a first-pass bolus imaging phase after Gd-CA, where the initial passage of the contrast agent is through the cardiac system. This method is based on rapid T1-weighting imaging with multiple images as a function of time, which allows a change in signal intensity, which is due to Gd atoms spreading from the blood circulation to the whole myocardium. This method shows the viable myocardium as hyperintensity areas, since the Gd atoms enter the remote and normal myocardium faster than from ischemic and scar areas, which are, therefore, seen as hypointensity areas [25]. However, myocardial perfusion is related linearly to the concentration of the CA, which prevents its use as an absolute quantitation method for myocardial perfusion [25]. Moreover, myocardial perfusion can be evaluated without the use of Gd-CA and these perfusion methods are arterial spin labeling [26] and blood oxygen level-dependent contrast [11], which can show the capillary flow in the viable myocardium [10].

**Figure 2:**
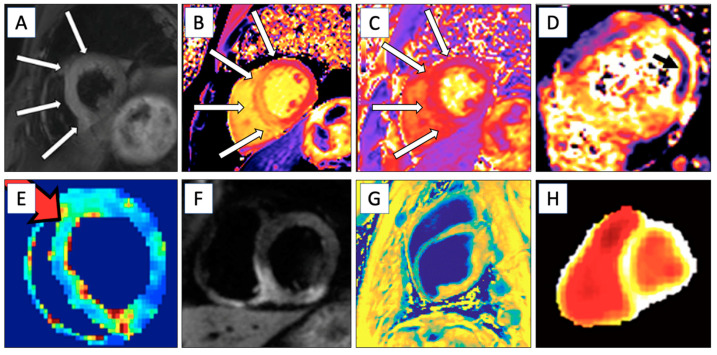
Different cardiac magnetic resonance acquisitions. (**A**) Late gadolinium enhancement image, T1 mapping (**B**), and T2 mapping (**C**) of an acute anteroseptal myocardial infarction (white arrow; adapted from Emrich, T et al., 2021 [42]). (**D**) Lateral infarct with intramyocardial hemorrhage seen in T2* mapping (black arrow; adapted from Ahmed N et al., 2016 [43]). (**E**) T1ρ image form anteroseptal infarct (red arrow) in a pig (Ylä-Herttuala E et al., Unpublished data, 2024). (**F**) Myocardial scar can be seen in the anterior septum, imaged with magnetization transfer imaging (adapted from López, K et al., 2020 [44]). (**G**) Inferior wall edema is seen in diffusion-weighted imaging 2 days after a myocardial infarction (adapted from Kociemba, A. et al., 2013 [45]). (**H**) Hyperpolarized cardiac imaging from a healthy person (adapted from Peder et al., 2023 [46]). Images (**A**–**D**) and (**F**–**G**) were modified (only part of the image was selected) by CC BY 2.0 or 4.0. https://creativecommons.org/licenses/by/.

At about 10–20 min after Gd injection, the late gadolinium enhancement (LGE) method is acquired [27]. LGE is the current gold standard for the determination of ischemic and scar areas (Figure 2A) in the myocardium [24,27]. The LGE method is based on the inversion recovery (IR) technique, where an normal and remote myocardium MR signal is nulled upon waiting for a specific period of time, the so-called inversion time, after the IR [27]. This creates more contrast between ischemic and scar tissue compared to the remote and healthy myocardium and therefore makes it easier to determine ischemic and scar tissue in the myocardium [27]. However, since the left ventricle (LV) blood pool has also Gd atoms in it, it is sometimes difficult to distinguish the hyperintensity areas and blood pool in the LV. One method to help to increase the contrast between ischemic and scar areas from the blood pool in LGE is to add T2 preparation between the IR and readout sequence [11,28,29].

From LGE images, the visual determination and quantitative measurement of ischemic and scar areas can be performed. The clinical cardiac MRI protocol typically includes T1 mapping acquired before Gd injection. If the extracellular volume (ECV) is to be measured, additional T1 mapping needs to be acquired after the Gd injection. Using these images and knowing the hematocrit value of the blood or using a standard correction value, the ECV of the heart can be calculated. This calculation provides additional information about the myocardial tissue composition, helping to quantify the extent of extracellular space expansion, which is indicative of fibrosis and other pathological changes in the myocardium [11,12,30]. It has been shown that the ischemic and scar tissue areas have higher ECV values (54 ± 1%) compared to the remote myocardium (29 ± 2%) [31].

### 4.3. Conventional Relaxation Time Methods in Myocardial Ischemia, Scar, and Viability Imaging

The characterization of myocardial tissue can be achieved without Gd-CA. These techniques are based on the assessment of changes in the intrinsic water properties within the myocardial tissue [10]. Conventional endogenous MRI methods to create contrast inside the myocardium include longitudinal T1 relaxation [24,32,33] and transversal T2 relaxation [32,34]. Generally, T1-weighted images are used for anatomical imaging and T2-weighted images for edema and transient ischemia imaging [35]. Both T1 and T2 relaxation methods can be also performed using mapping techniques. In these methods, several relaxation-weighted images are collected, and the signal intensities from each pixel in each weighted image are then fitted with linear or non-linear functions on a pixel-by-pixel basis to calculate the relaxation time in each pixel. These data are then used to create a relaxation time map [34,36,37]. From these calculated relaxation time maps, changes within the myocardium caused by ischemic, scar, or other cardiovascular injuries can be analyzed both visually and numerically. These techniques provide a non-invasive way to assess the myocardial tissue properties and detect pathological changes without the need for CAs [34,36].

The T1 relaxation time increases in areas of ischemia and scar tissue (Figure 2B). This change in the T1 relaxation time can be used as a biomarker to identify and assess the extent of myocardial damage [31,38]. The T1 relaxation times are even higher in the acute phase of scar tissue development than in the chronic phase. During the acute phase, edematous and necrotic tissue contributes to elevated T1 values. As the scar matures, these tissue areas are replaced by smaller amounts of enlarged extracellular collagen [39]. T2 relaxation maps (Figure 2C) are suitable to determine the acute phase of the ischemic area since it is sensitive to edema [18]. However, T2 relaxation time methods suffer from a poor contrast-to-noise ratio, which limits their use to determine the area of acute ischemic injury [40].

The T2* relaxation time method accounts for magnetic field inhomogeneities and magnetic susceptibility differences, along with the usual T2 properties [18]. A T2* relaxation map (Figure 2D) is more sensitive to magnetic susceptibility changes than a normal T2 relaxation map and is used to determine an iron overload in the myocardium due to iron accumulation or a myocardial hemorrhage in the acute phase [18,41].

### 4.4. Rotating Frame Relaxation Time Methods for Ischemic and Scar Imaging

One advanced method to image myocardial ischemia and scars with mMRI without using a CA is the longitudinal rotating frame relaxation time method, where the relaxation occurs during the radio frequency (RF) pulse and not after, as in conventional T1 and T2 relaxation time methods [47,48].

The T1ρ (T1 rho; Figure 2E) mMRI method is used to detect subtle changes in tissue composition and provides enhanced contrast and sensitivity. This can be used to detect myocardial ischemia and scarring. Unlike the conventional T1 and T2 relaxation times, which measure how quickly the spins return to equilibrium after being disturbed, T1ρ measures relaxation during the RF pulse. Relaxation in the rotating frame occurs along the time-dependent effective magnetic field, or spin lock field, which is a vector sum of the external magnetic field and the magnetic field of the RF pulse. There are many techniques used to create T1ρ contrast in MRI, and the most common ones are the continuous wave (CW) RF pulse method [49] and adiabatic RF pulse method [48]. Enhanced T1ρ relaxation times with the CW T1ρ method have been shown in increased ECV, ischemic, fibrotic, and scar areas [24,47]. A correlation between increased T1ρ values and LGE findings has been observed in studies involving mice [24,50], pigs [40], and humans [51]. One limitation in the use of the T1ρ relaxation time method in clinical MRI protocols is the relatively high specific absorption rate (SAR), which causes the heating of the tissue [24].

**Figure 3:**
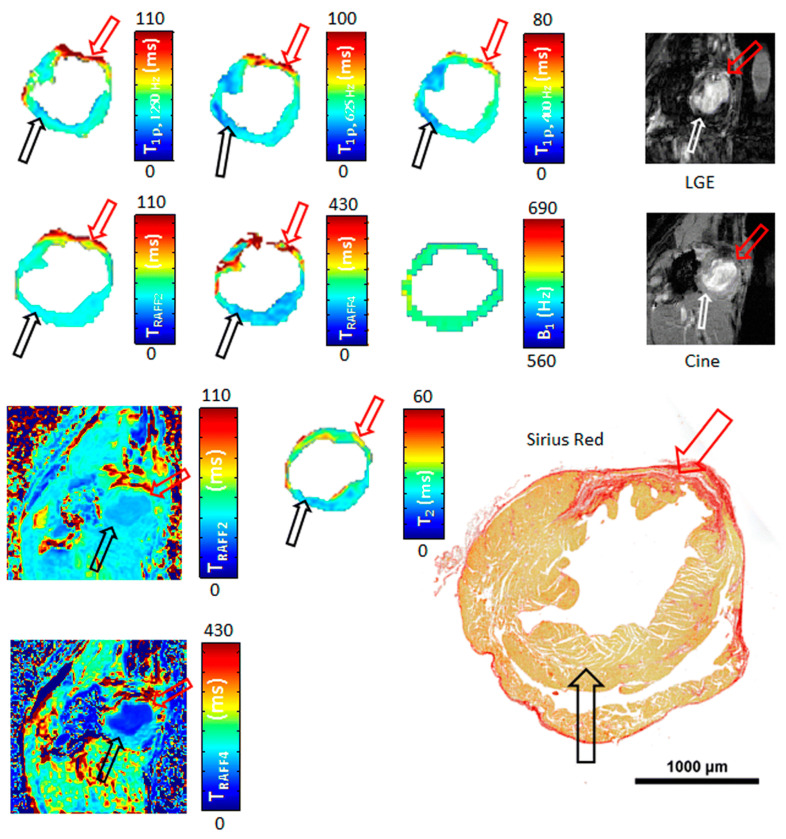
Relaxation along a fictitious field (RAFF) map with late gadolinium enhancement (LGE) images, cine, and a corresponding histology section stained with Sirius Red from an infarcted mouse heart 21 days after an infarction. The red arrow indicates the infarct area, and the black/white arrow shows the control area. Image adapted from Ylä-Herttuala et al., 2019 [10] by CC BY 4.0. https://creativecommons.org/licenses/by/4.0/

To address these SAR values, relaxation along a fictitious field (RAFF) in the n^th^ rotating frame (RAFFn; Figure 3) has been designed [24,52,53]. RAFFn relaxation occurs along a fictitious magnetic field, which is produced by a fast sweep of the effective RF field to achieve sub-adiabatic conditions in the target tissue [52,53]. The advantages of the low SAR values are seen when RAFF is used in the higher-rank (n) rotating frames (RAFFn), since the higher the rank in the RAFF pulse, the lower the flip angle needed [52,53]. The contrast between fibrotic myocardial infarction and remote areas has been demonstrated with the RAFFn technique in mice [24,54]. The RAFFn values were higher in histologically proven fibrotic areas after an induced myocardial infarction, and the results also corresponded to the CW T1ρ and LGE findings [24].

Rotating frame relaxation time methods, such as T1ρ and RAFFn, are used during the RF pulse, which operates in a different chemical exchange or frequency range (0.1–10 kHz) compared to conventional relaxation time methods, which are performed after the RF pulse at the Larmor frequency (10–500 MHz). Therefore, rotating frame relaxation time methods are sensitive to slow molecular motions and low-frequency interactions between macromolecular, bounded water and free water molecules (correlation time regime 10^−1^–10^−5^ s), which occur in the fibrotic, granulated, collagen-based matrix and scar tissue [24]. Comparing rotating frame relaxation time methods to conventional T1 and T2, these conventional MRI methods are also sensitive to the mean degrees of freedom of water molecules together with slow molecular motions, and they are both non-selective and sensitive to a different range of exchange and correlation times (around 10^−6^–10^−9^ s). Additionally, since the rotating frame relaxation time methods are performed during the RF pulse, the choice of the RF pulse and the RF pulse parameters can be modified almost an infinite number of times. Thus, finding the correct combination of RF pulse and its parameters is crucial to accurately determine the molecular levels of ischemic and scar tissue in the myocardium.

### 4.5. Magnetization Transfer Method for Ischemia, Scar, and Viability Imaging

Magnetization transfer (MT; Figure 2F) is an MRI method that determines restricted water–macromolecular proton interactions by saturating the free water pool proton signal [54]. Consequently, MT is an effective technique for the imaging of macromolecules in the myocardium [55]. This is achieved by using the magnetic resonance (MR) preparation of off-resonance RF pulses while acquiring the MR signal with an on-resonance readout sequence [55]. The MT method has enhanced the contrast difference between a myocardial infarction and remote myocardium [55,56]. Additionally, MT has provided a contrast difference between the blood and healthy myocardium [55,57]. The MT method can be used together with T1 weighting and T1 mapping to reveal myocardial ischemic injuries within the rest of the myocardium without using a CA [55]. With MT preparation, it is possible to find the best off-resonance frequencies and lengths for MT preparations and, alongside this, to vary the parameters of the on-resonance readout sequence, such as using two different RF powers in it [58], using different flip angles [59], or enhancing the contrast between the myocardial injury and remote myocardium [55].

### 4.6. Diffusion Techniques for Ischemia, Scar, and Viability Imaging

Recently, diffusion-weighted imaging (Figure 2G) has been implemented in molecular cardiac imaging, as water diffusion is increased in ischemic areas compared to the rest of the myocardium [60]. Diffusion changes in ischemic and scarred areas are due to the increase in the ECV and the decrease in functional cardiomyocytes, which alter the orientational structure of the myocardium [11]. Moreover, there is a technique called diffusion tractography that measures the orientation of cardiomyocytes [14]. This technique can be used, for example, after an ischemic event to determine disturbances in the helical fiber architecture [11]. However, this novel imaging technique is currently highly time-consuming and, therefore, not yet feasible for routine clinical workflows.

### 4.7. Hyperpolarization MRI for Ischemic, Scar, and Viability Imaging

Real-time metabolic activity can be determined with a novel MRI technique called hyperpolarized MRI (hMRI; Figure 2H) [61]. The basic idea of hMRI is that it produces an approximately 10,000-times stronger MR signal compared to conventional MR spectroscopy for a short period. This increased signal allows for the accurate determination of low natural abundances of metabolic compounds in both the normal and ischemic myocardium [61]. This subject will be further discussed in Section 5.1 and Section 5.2. 

## 5. Molecular MRI Contrast and Imaging Agents

The ideal pharmacokinetics of the molecular probe involve rapid localization at the target site and the swift clearance of the non-specific background, allowing for pre- and post-injection imaging within a single session. Additionally, the size of the probe is crucial. A probe with a molecular weight <10 kDa is usually small enough to quickly penetrate the extracellular space and be efficiently cleared from the body [62]. In contrast, nanoparticles and macromolecules generally require more time to reach their targets and are often associated with prolonged excretion in the urine [62]. Moreover, the probes should be well tolerated. It has been shown that gadolinium-based contrast agents (GBCA) are overall well tolerated. In a study consisting of 72,839 GBCA-enhanced MRI cases, the acute adverse event rate (AAE) was 0.36%, with a minority of severe adverse effects at 0.033% [63]. Moreover, allergic-like AAEs were less likely than physiologic AAEs (29% vs. 71%). There was further marginal evidence that a higher GBCA volume was associated with a higher AAE incidence (OR = 1.02, *p* = 0.05). Beyond this, nephrogenic systemic fibrosis (NSF) is a form of Gd-associated toxicity that can occur in patients with poor renal function who are exposed to GBCAs. However, the risk of NSF is small, even in patients with a glomerular filtration rate of less than 30 mL/min/1.73 m^2^, with an incidence rate of 0.07% [64].

Conventional Gd chelates offer sensitivity that is superior to that of iodinated CAs, albeit less than that of radiotracer and fluorescence techniques [65]. Targeted CAs hold promise in enhancing the detection and characterization of molecular changes associated with various cardiovascular diseases, thereby enabling early diagnosis, treatment monitoring, and personalized medicine approaches. To enhance the sensitivity, it is possible to use highly expressed molecular targets like collagen-specific MRI targets [66] or use novel MRI agents that have significantly higher relaxivity than conventional Gd chelates. Moreover, atherosclerosis can be visualized using Gd-containing liposomes, lipoproteins, micelles, and superparamagnetic iron oxide magnetic nanoparticles (MNPs) [65]. Furthermore, Winter et al. have shown that targeted paramagnetic nanoparticles can be used to detect early atherosclerosis in a rapid manner, to deliver an antiangiogenic drug, and to quantitatively assess the neovascular response [67]. For these reasons, mMRI offers valuable assistance in identifying precise drug-targeting sites and determining the optimal timing for the administration of novel treatments (for example, specific anti-inflammatory therapy), thereby possibly enhancing the therapeutic outcomes [68].

### 5.1. Imaging Myocardial Perfusion

Myocardial perfusion can be imagined with Gd, as previously described. Myocardial perfusion can also be studied with [1-^13^C] hMRI, and such study is performed similarly as with Gd first-pass bolus [25]. Since there is no natural abundance of [1-^13^C] in the body—for example, in an ischemic myocardium—the perfusion signal can be seen better with [1-^13^C] hMRI than GdCA because there is no signal coming from underlying structures [25]. Additionally, [1-^13^C]-urea and [1-^13^C]-pyruvate molecules can be used in hMRI as co-polarized and co-administrated molecules, providing a one-time injection to determine cardiac perfusion and metabolism at the same time [25,69]. This would be valuable in assessing myocardial viability, with advances in the imaging time and radiation-free methods as compared to PET techniques [25].

### 5.2. Imaging Cellular Hypoxemia and Metabolism

hMRI (Figure 2H) is mostly used together with the dynamic nuclear polarization (DNP) method, where [1-^13^C]-labeled pyruvate is injected and then its downstream metabolism is followed. Pyruvate plays a central role in cardiac energy metabolism as it is the final product of glycolytic glucose breakdown [61]. Under normal aerobic conditions, pyruvate is converted into acetyl-CoA and CO2/bicarbonate molecules [61]. However, under anaerobic conditions, energy is produced through lactate formation via lactate dehydrogenase [61]. Thus, DNP imaging reflects the energy homeostasis and could be used to image cardiac ischemia [61]. Although hMRI has been used mostly in humans for oncology [70], there have also been studies focused on cardiac imaging [71]. However, cardiac hMRI has primarily been used in experimental ischemic animal models [70,72,73,74].

Increased lactate levels and decreased bicarbonate levels have been observed in pig myocardial reperfusion studies and ex vivo infarction studies. These findings indicate the rapid metabolic changes occurring in the acute and chronic ischemic areas of the myocardium following reperfusion [74,75,76,77]. Additionally, when the bicarbonate levels returned to normal after reperfusion, the LV wall motion was preserved; however, the LGE results remained unchanged [73]. Furthermore, LV systolic dysfunction in rats has been correlated with a reduction in Krebs cycle flux [78]. It is also possible to image pH changes in the myocardium using mMRI [61]. This is significant from an ischemic perspective since ischemic areas exhibit decreased pH values due to increased glycolysis and the resulting elevated production of intracellular protons and lactic acid [61,79,80].

The biology of the myocardium after ischemia and revascularization is intriguing due to the distinct pathophysiological differences between these two phenomena. These differences manifest in the integrity of the cell membrane in cardiomyocytes and the activation of glucose metabolism in the hibernating viable myocardium. In contrast, the non-viable myocardium lacks these biological properties [25]. This can be seen as hypo-contractility in the hibernating myocardium with imaging, which can be improved with revascularization [25]. Therefore, the imaging of myocardial perfusion, contractility, and the metabolism of oxidative and glycolytic carbohydrates with hMRI and [1-^13^C]-labeled pyruvate almost at the same time would give new information about the biology of the hibernating myocardium [25].

### 5.3. Protein-Targeted Gadolinium-Based Contrast Agents

Gd-CA gadofosveset (trade names Ablavar, Vasovist) has been previously used for contrast-enhanced magnetic resonance angiography to visualize abdominal or limb vessels. It also has albumin-binding properties. Acute ischemia leads to myocardial endothelial damage, causing plasma albumin to leak into the myocardial extravascular space. The vascular permeability following a myocardial infarction has been studied in mice [81]. It has been shown that gadofosveset enables the detection of changes in myocardial permeability, allowing for differentiation between the acute and chronic phases post-myocardial infarction [81]. The usability of gadofosveset in diagnosing CAD has been studied in humans. Twenty-six patients underwent angiography and gadofosveset MRI. The study found that patients with coronary stenosis ≥70% showed increased signal enhancement on gadofosveset-enhanced MRI downstream of the stenosis, suggesting higher endothelial permeability in these lesions [82].

Fibrosis consists mostly of type I collagen. The first type I collagen-targeted MRI contrast agent, EP-3533 (comprises a 16-amino-acid peptide with a 10-amino-acid disulfide bridge cyclic core conjugated to three Gd moieties), was able to show high contrast for a fibrotic scar versus viable myocardium in a mouse model [66]. The washout time for EP-3533 is relatively long in regions of post-infarction scarring (mean, 194.8 min ±116.8 SD) and longer than in a normal myocardium (mean, 45.4 min ± 16.7 SD) [83]. No human studies have been performed so far with EP-3533.

## 6. Comparison and Integration of mMRI with PET

PET is an imaging method that utilizes radioactive tracers to detect and measure biological activity in both healthy and, more crucially, diseased tissue. PET offers many different molecular imaging methods for the detection of fragile plaques, ischemia, and post-infarction changes in the myocardium. In humans, these methods are often first used in the carotid arteries, offering a high atherosclerotic burden without much attenuation or movement. PET provides a spatial resolution of about 5 mm in a stable environment; the coronary arteries and the myocardium, however, are in constant movement. In contrast, MRI typically achieves a spatial resolution of 1–2 mm [84]. With high-resolution MRI techniques, the spatial resolution can be even finer, often less than 1 mm [85]. Additionally, the temporal resolution is significantly higher in MRI (20–50 ms) compared to PET (5 s–5 min) [84]. However, ultrasound has the highest temporal resolution (<5 ms) [84]. As combined PET/MRI systems become more common, they offer new possibilities for true multimodality molecular imaging. This integration significantly enhances both the spatial and temporal resolution. PET/MRI provides the high soft tissue contrast and spatial resolution of MRI, coupled with the molecular and functional imaging capabilities of PET. This allows for the more precise localization and characterization of pathological processes. Additionally, the simultaneous acquisition reduces misalignment errors caused by patient movement between separate scans, further improving the accuracy and reliability of the imaging.

### PET Imaging Tracers

Both atherosclerosis-related inflammation and fragile plaques specifically are avid on fluorodeoxyglucose (FDG) PET [86]. The FDG avidity is due to the inflammation process, as well as the neovasculature and loose extracellular matrix [87]. However, the adjacent myocardial FDG uptake may make coronary uptake difficult to analyze. Another radiopharmaceutical that has been widely studied with CAD is ^18^F (fluorine) or ^18^F-NaF (fluorine-labeled sodium fluoride), due to the presence of microcalcification in the plaque. However, it still is not established whether there is a place for either FDG or NaF in risk stratification or intervention guidance [88]. Another interesting prospect is the utility of macrophage-targeted methods, such as somatostatin receptor or folate receptor imaging in atherosclerosis [89,90].

The assessment of viability, on the other hand, is well established, with articles from the 1980s stating that the imaging mismatch of perfusion and metabolism shows a viable yet hibernating myocardium [91]. Perfusion can be assessed with modern PET tracers. ^82^Rb (rubidium), ^15^O (oxygen-labeled water), and ^13^N (nitrogen-labeled ammonia) are the most used PET perfusion tracers. They share a limitation, namely the very short radioactive half-life of mere minutes. Novel ^18^F-flurpiridaz makes the PET imaging of perfusion more accessible due to its longer half-life of 118 min. Still, FDG remains the gold standard of metabolism, showing high sensitivity and moderate specificity for the recovery of regional function [92]. A viable myocardium shows glucose metabolism in the form of FDG uptake, whereas absent or markedly reduced uptake indicates scarring. While retrospective studies have indicated lower mortality rates after the revascularization of the viable myocardium compared to those who do not undergo revascularization, a randomized controlled trial with chronic ischemic HF patients did not show a statistically significant mortality benefit compared to the standard of care [93,94].

Another new and interesting tracer is fibroblast-activating protein inhibitor (FAPI). FAPI can be labeled with either ^68^Ga (gallium) or ^18^F, making it either a generator-based or transportable tracer. After a myocardial infarction, fibroblasts are activated to promote scar formation. In animal models, the activation peak is reached six days after the infarction, and the highest number of fibroblasts is found in the border zone of the infarcted area [95,96]. This may be due to remodeling and may serve as a new therapeutic target. Studies in patients with ST elevation myocardial infarction have shown that baseline FAPI-PET is better than cardiac MRI in predicting remodeling after a myocardial infarction [97,98]. The usability of [1-^13^C]-labeled pyruvate has been discussed in the previous section.

## 7. Conclusions

Significant advancements have been made in the past decade in imaging myocardial ischemia, scarring, and viability. The introduction of new mMRI sequences and target-specific CAs has revolutionized the molecular imaging of these conditions. In clinical settings, mMRI has the potential to enhance the imaging accuracy and enable the earlier detection of cardiovascular diseases. These methods could transform the management of myocardial infarction patients by improving patient stratification, enabling personalized treatments, and evaluating the treatment efficacy. Additionally, mMRI techniques have the potential to identify patients at higher risk for adverse cardiovascular events.

A key advantage of MRI is its non-invasive nature and absence of a radiation burden, making it safe for patients. However, extensive research is still required at both the preclinical and clinical stages to further develop these imaging methods and CAs. The combination of MRI and PET could further facilitate the imaging process and provide new insights into cardiac ischemia and its pathophysiology.

## Figures and Tables

**Table 1:** Summary of cardiac magnetic resonance (CMR) methods used to evaluate ischemia, scarring, and viability.

CMR Technique	Information Obtained	LGE	Examples of Diseases Studied	Figure
T1 Mapping	Identifies myocardial tissue properties like fibrosis. Extracellular volume can be measured if LGE is used.	No/Yes	Myocardial infarction, myocarditis, amyloidosis, etc.	Figure 2B
T2 Mapping	Evaluates edema, suitable for detection of acute ischemic areas.	No	Acute ischemia, myocarditis, etc.	Figure 2C and Figure 3
T2* Relaxation	Myocardial hemorrhage in the acute phase.	No	Ischemic hemorrhage and iron overload	Figure 2D
Late Gadolinium Enhancement	Determines ischemic and scar areas and visualizes damage from healthy myocardial tissue. Perfusion imaging.	Yes	Ischemic heart disease (fibrosis), inflammation, etc.	Figure 2A and Figure 3
T1ρ Imaging	Detects subtle changes in tissue composition, with enhanced contrast and sensitivity for ischemia and scarring.	No	Fibrosis	Figure 2E
RAFFn Imaging	Analyzes molecular levels of ischemic and scar tissue with low SAR values, suitable for fibrotic areas.	No	Fibrosis	Figure 3
Magnetization Transfer Imaging	Determines water–macromolecular proton interactions and enhances contrast between myocardial infarction and remote myocardium.	No	Myocardial infarction	Figure 2F
Diffusion-Weighted Imaging	Detects areas of myocardial infarction by identifying regions with restricted water diffusion, which correspond to ischemic injury. Also detects myocardial fibrosis with altered diffusion properties.	No	Ischemic heart disease	Figure 2G
Hyperpolarized MRI (hMRI)	Determines real-time cardiac energy metabolism with hyperpolarized [1-^13^C]pyruvate.	No	Ischemic heart disease	Figure 2H

RAFFn = relaxation along a fictitious field (RAFF) in the nth rotating frame, SAR = specific absorption rate, LGE = late gadolinium enhancement.
